# Driving Innovation to Support Pupils with SEND Through Co-Production in Education and Research: Participatory Action Research with 22q11.2 Deletion Syndrome Families in England

**DOI:** 10.3390/bs15010022

**Published:** 2024-12-30

**Authors:** Michelle Jayman, Sophie Edmonds, Maria Gudbrandsen

**Affiliations:** 1 School of Psychology, University of Roehampton, London SW15 4JD, UK; maria.gudbrandsen@roehampton.ac.uk; 2 NHS Surrey and Borders Partnership, Leatherhead, Surrey KT22 7AD, UK; sophie.edmonds@sabp.nhs.uk

**Keywords:** special educational needs and disabilities, 22q11.2 deletion syndrome, mainstream schools, pupil support, mental health and wellbeing, inclusion, participatory action research

## Abstract

Children and young people (CYP) with special educational needs and disabilities (SEND) comprise over 1.6 million pupils in classrooms in England. However, evidence suggests pupils’ learning and wellbeing needs are often missed or unmet and legislation designed to increase families’ decision-making in education provision has not been translated into practice. The current participatory action research study investigated the perceptions and experiences of a specific population of SEND pupils in mainstream schooling—CYP with 22q11.2 deletion syndrome (22q). Participants included existing and previous mainstream pupils and their parents (n = 8 parent−CYP dyads). Data were collected through semi-structured interviews, and a hybrid inductive-deductive thematic analysis was conducted. Five superordinate themes were generated: minding the gaps in school support; my mental wellbeing story; power and influence; getting it wrong: failing CYP and families; and getting it right: from surviving to thriving. Findings provided authentic insights into the lived experiences of support for CYP with 22q which resonate with the wider SEND population. These findings can help to inform more inclusive practice in mainstream settings. An affirmative model which places SEND pupils and parents at the heart of meaningful reform is urgently needed in schools. Collaborative work among all key stakeholders is paramount to ensure that strategies are genuinely co-produced, co-owned and robustly evidence-based.

## 1. Introduction

“The SEND system is broken”—this was the stark conclusion drawn by independent researchers commissioned to review current provision in England (ISOS Partnership, 2024, p. 3). The statement reflects the general consensus among key stakeholders a decade on from landmark reforms introduced through the Children and Families Act 2014 (Atkinson et al., 2024) and highlights the plight of over one and a half million pupils in England identified with SEND who face significant delays in having their needs identified and receiving appropriate support (GOV.UK, 2021). According to the National Audit Office (2019), approximately 40 per cent of children and young people (CYP) are identified with SEND during their school years. While the effective, consistent, and joined-up approach envisioned by the 2014 reforms has not been realised (HM Government, 2023), the number of CYP with an Education and Health Care Plan (EHCP)^1^ has increased by 140% in the decade since their inception (from 240,183 in 2014/15 to 575,973 in 2023/24) (DfE, 2024). Notably, this surge in demand for SEND support is more prevalent in England than in other comparable European nations (ISOS Partnership, 2024). Proposals emanating from a 2022 government review of SEND echoed previous pledges to improve systemic issues and to deliver a single national system with “consistent standards for how needs are identified and met at every stage of a child’s journey across education, health and care” (HM Government, 2022, p. 14). This mantra for reform has been reiterated by the present UK government alongside a commitment to improving inclusion and expertise in mainstream schools (Atkinson et al., 2024). The current Participatory Action Research (PAR) champions the core principle of inclusivity in education and in research and, crucially, places CYP with SEND and their families at the heart of driving meaningful and effective change. This is explored through the experiences of a subpopulation of SEND pupils, individuals with 22q11.2 Deletion Syndrome; however, the authors posit that the research findings will resonate beyond this group to a larger SEND population and, therefore, offer broader insights for rethinking and reshaping inclusion strategies.

### 1.1. The State of the Nation: SEND Education

A report by the Institute for Fiscal Studies(Farquharson et al., 2024) branded the escalating number of CYP receiving SEND support as one of the costliest challenges facing mainstream schools in England. Despite increased government assistance to reflect growing need, this has failed to keep pace with the rapid growth in school expenditure (Sibieta, 2024). In one survey, 99% of school leaders reported that funding for pupils with SEND was insufficient to meet individual needs (Norden, 2024). Unsurprisingly, CYP with SEND have shown consistently poorer outcomes than their peers at every level of education (HM Government, 2022). In 2023, just 8 per cent of pupils with an EHCP met the expected standard in reading, writing and Maths (combined) at the end of primary school (age 11) compared to 60 per cent of their non-SEND peers (GOV.UK, 2023); while at the end of compulsory secondary education (age 16), less than a third (30 per cent) of pupils with SEND achieved a Grade 4 or higher in GCSE English and Maths compared to almost three quarters (72 per cent) of those without SEND (GOV.UK, 2024). Strikingly, in some regions in the north of England, only 27 per cent of SEND pupils achieved a Grade 4 or above in GCSE English and Maths, while in Inner London, this figure was significantly higher at 39 per cent. These geographical discrepancies expose a fragmented and inequitable system but also illustrate the relative success that some SEND pupils can achieve despite a national funding crisis.

### 1.2. More than Academics: Schools for Wellbeing

The primary function of mainstream schools is generally agreed to be education. Nonetheless, it is broadly acknowledged that pastoral care and managing CYP’s emotional and social behaviours are firmly rooted in the remit of educators (Hurry et al., 2021). Schools provide strategic settings for nurturing socio-emotional competencies, delivering evidence-based wellbeing interventions and identifying pupils at risk of experiencing difficulties (Mansfield et al., 2021). From an ecological stance, educators are well placed to offer such provision, now enshrined in the statutory responsibilities of English schools (DfE, 2019). Supporting CYP’s wellbeing—both explicitly (for example, teacher-led interventions) and through the “hidden curriculum” (the values and ethos that pupils are exposed to)—can lead to improvements in pupils’ school connectedness, academic engagement and performance (Hurry et al., 2021). Nonetheless, school staff are expected to accommodate an increasingly wide spectrum of need, while lack of clarity in their role and inadequate training has placed an unmanageable burden on educators who are ‘buckling under the strain’ (Education Support, 2023). Consequently, many pupils are not receiving the wellbeing support they need which, in turn, negatively impacts on their learning (Lindorff, 2020). Furthermore, wellbeing issues are often overlooked for pupils with SEND when the overriding focus is on learning outcomes (Boesley & Crane, 2018). Socio-emotional difficulties may also go undetected due to diagnostic overshadowing—when certain behaviours are misattributed to the primary diagnosis rather than co-occurring (Children and Young People’s Mental Health Coalition, 2019). In combination, these factors present significant barriers to pupils receiving timely and appropriate support and may hinder CYP’s willingness to disclose any issues, further exacerbating their difficulties (Cerebra, 2023).

In 2021, more than half (56 per cent) of 6- to 16-year-olds with SEND in England had a probable mental disorder compared with 12.5 per cent of their non-SEND peers (Newlove-Delgado et al., 2021). Moreover, behavioural, attentional and emotional difficulties appeared to worsen for CYP with SEND in the wake of the COVID-19 pandemic while levels remained relatively stable for other groups (Kuhn et al., 2024). Conversely, some parents reported that their SEND children were happier and less anxious during lockdown restrictions due to not having to physically attend school (Parentkind, 2020). In related research, Barnes and Harrison (2017) found a statistically significant difference for school wellbeing between CYP with and without SEND (those with demonstrated lower levels). Negative school experiences are highlighted by common instances of bullying: in one survey, 29 per cent of pupils with SEND reported “frequent” bullying; with CYP with autism or social, emotional and mental health (SEMH) difficulties identified as the group most likely to be afflicted (Anti-Bullying Alliance, 2023). Worryingly, some pupils with SEND struggle to articulate their experiences so instances can go unreported, allowing bullying to persist unchallenged (Sanderson, 2024).

The aims of inclusive education are to create universally optimal conditions for both learning and socio-emotional development (Ainscow & César, 2006). Nonetheless, research by Goldan et al. (2022)revealed that by the end of primary education, pupils with SEND reported less satisfaction with school, as well as their friendships and life in general, compared to their non-SEND peers. These findings aligned with previous research (Swift et al., 2021) and were partly attributed to persistent stigmatisation and negative attitudes towards SEND within the school environment. Similarly, in a study with older children (aged 11–15), Skrzypiec et al. (2015) discovered that pupils with SEND were more likely to have low subjective wellbeing (languishing rather than flourishing) and experience mental health difficulties compared to their non-SEND counterparts. The post-pandemic school experience for CYP with SEND is deeply concerning, given evidence suggesting higher numbers of pupils are presenting as withdrawn, alongside increased rates of reported suicidal thoughts and self-harm (Ashworth et al., 2024). These findings highlight the urgency for improved support and interventions to ameliorate the school experience and wellbeing of pupils with SEND including the subpopulation of CYP who are the focus of the current study.

### 1.3. Legacy and (New) Directions for Positive Change in SEND Provision

The pivotal role of education in creating a more inclusive society is staunchly advocated, with mainstream schools well positioned to meet the needs of a large proportion of the SEND population (DfE & DoH, 2015). However, contrary to this egalitarian stance, evidence strongly suggests that the legacy of previous SEND reforms is a markedly less inclusive education system. A deficit-based understanding of CYP’s needs remains the dominant discourse in schools, while limited focus on SEND and inclusion in teacher training and continuous professional development (CPD) engenders poor understanding of CYP’s needs and how to provide appropriate support (ISOS Partnership, 2024). Qualitative findings (Warnes et al., 2021, p. 31) revealed that some staff considered SEND responsibilities to be an “onerous adjunct to an already stressful ‘regular’ teaching role”. However, other research has shown that teachers’ experiences of working with SEND pupils are linked to more positive attitudes and their ability to foster a more inclusive classroom (Leatherman & Niemeyer, 2005). Clearly, healthy attitudes towards SEND work and educators’ conviction in the quality of their training are prerequisites for regular inclusive practice. However, evidence suggests a paucity of training on the diverse needs of pupils (Bosanquet & Radford, 2019; Sharples et al., 2018; Warnes et al., 2021) compounded by the lack of clear accountability systems which thwart efforts to formally challenge non-inclusive practices operating in schools (ISOS Partnership, 2024). Related research showed that SEND co-ordinators (SENDCos) are particularly afflicted by time poverty and perceived lowly status (Curran, 2019; M. D. Smith & Broomhead, 2019). In one study conducted during the COVID-19 pandemic, respondents admitted to feeling unable to ensure that provision for vulnerable pupils was adequate, which had a negative impact on staff’s own mental wellbeing (Boddison & Curran, 2021). Another study, involving 15 SENDCos, revealed that most did not self-identify as an “expert” in additional needs, in sharp contrast to the majority of SEND families who considered them to be an authority in their field (M. D. Smith & Broomhead, 2019).

### 1.4. Reconceptualising the Expert: My EHCP—Where Is My Voice?

In 2019, the House of Commons Education Committee (HCEC) concluded, “More needs to be done to include children and young people in the writing of their [EHC] plans and decision-making about the support they receive” (HCEC, 2019, p. 87). However, there is a dearth of evidence to show that this directive has been translated into practice. One survey found that only a quarter (25 per cent) of parents were satisfied with the EHCP their child had received, while 50 per cent expressed dissatisfaction with the whole system (National Autistic Society, 2021). In other research, parents reported “an intimidating and overwhelmingly difficult process” fraught with systemic barriers which negatively impacted on their mental health (Keville et al., 2024, p. 1). One systematic literature review (Ahad et al., 2022) concluded that many parents were either not involved in their child’s EHCP or were reliant on the professionals’ (e.g., SENDCos and educational psychologists) discretion—and if they were consulted, parents’ contribution was often strongly directed by others. CYP themselves had very limited involvement in developing plans, despite being the main beneficiary. Clearly, the voices of pupils with SEND and their parents should be at the centre of assessing individual needs and are central to an authentic understanding of CYP’s wider educational experiences. Article 12 of the United Nations General Assembly (1989) gives CYP the right to express their views on matters affecting them—including their education. Nonetheless, research concerning CYP has relied heavily on proxy views, and even more so for CYP with SEND. Increasingly, however, CYP are recognised as the “actor in” research rather than the “object of” research (Mason & Hood, 2011); this includes studies with very young children (Dockett et al., 2009), looked-after children (Bradwell et al., 2011), marginalised youth (Moore et al., 2013) and CYP with SEND (Dimitrellou & Male, 2019).

In one such study, Jordan et al. (2022) investigated the school experiences of secondary-aged pupils with SEND. Academic progress was perceived by CYP as the primary concern of staff, while sensitivity to pupils’ socio-emotional needs was lacking. For example, individual self-calming strategies (e.g., fidgeting) were interpreted by some teachers as disruptive. Bullying and peer rejection and not feeling involved or listened to by staff were factors identified as having a profound impact on pupil’s learning and wellbeing. Related research by Dimitrellou and Male (2019) found that although SEND pupils’ mainstream experiences varied according to their specific needs, notions of a positive school experience were universal—interesting lessons, well-managed classrooms, support from teachers and good relationships. Evidently, harnessing the voices of pupils with SEND is essential for improving inclusive practice; however, Dimitrellou and Male discovered that many CYP abstained from pupil voice activities (e.g., school council), believing their views would not be actioned and decision-making rested ultimately with adults (teachers). Other research has shown that CYP lack a sense of belonging in settings where their views are not sought, valued or acted upon which, in turn, impoverishes their psychosocial development and wellbeing (Woodhead & Brooker, 2008). Thus, pupil voice and school connectedness must be encouraged as both represent important protective factors for mental wellbeing (Halliday et al., 2018; McCabe et al., 2022).

### 1.5. Rationale, Framework and Aims for the Current Study

Despite repeated pledges for the national SEND system to be co-produced with families (HM Government, 2022), the evidence strongly suggests that even when their views are sought, CYP and their parents are often disempowered (Jordan et al., 2022; Keville et al., 2024), proper agency is lacking (Ahad et al., 2022), and consultation rarely leads into meaningful action (Dimitrellou & Male, 2019). A pupil with SEND appears to be doubly disadvantaged by the hierarchical nature of education and research—first by being a minor (Punch, 2002) and then by having an “impairment” (S. E. Jones, 2024). The current study aligns with an affirmative model of SEND, firmly rooted in the conviction, “nothing about us is without us” (Charlton, 1998), cited by S. E. Jones (2024, p. 9). Reflecting on their personal journey as a disabled pupil in mainstream education, Jones experienced the structural disadvantages inherent in a medical model of disability (with the onus on individual impairment) which serves to internalise notions of dependency and passivity. Frameworks underpinned by a social perspective (Oliver, 1983)have helped reshape public perceptions (and policies) related to disability, shifting the responsibility on society (and institutions) to adapt to individual needs. Crucially, as Jones points out, affirmative models of disability (Swain & French, 2000) generated by disabled people and borne from lived experience—strive beyond adaptive action and insist that all people with a disability and additional needs are recognised as valid citizens with agency and value and that individual differences should be celebrated. The present crisis in the SEND sector shows no signs of abating and fully understanding the challenges pupils with SEND and their families encounter in mainstream schooling is imperative to help revoke the legacy of failure. The overall aim of the current study was to capture the authentic voices of a subpopulation of SEND—CYP with 22q11.2 deletion syndrome (22q) and their parents, specifically to better understand the learning and wellbeing challenges experienced in mainstream education to inform more inclusive practice targeting pupils’ holistic needs. Although the current study concerns CYP with (22q), the impetus for the research was to help inform more inclusive practice for the wider SEND population. Furthermore, while the focus is on mainstream schools in England, the research area and study findings have value and relevance of international scope.

## 2. Materials and Methods

### 2.1. Design

In line with the study’s affirmative framework, a participatory action research (PAR) design—democratic, equitable, liberating and life-enhancing (Koch & Kralik, 2006) was used. This involves the co-production of research knowledge and recognises that different communities have the unique skills and expertise to best understand local needs through personal lived experiences (Lloyd-Evans et al., 2023). Moreover, it upholds the fundamental human right for individuals to contribute to decisions that affect them (Reason & Bradbury, 2008). In the current study, this refers to CYP with SEND, specifically with (22q). Research knowledge is generated for the purpose of social justice and its positive impact on everyday lives, not for its own sake (MacDonald, 2012). Pertinently, as Doucet et al. (2022) have pointed out, in research with disenfranchised youth, academics must be mindful of their own privilege and actively pursue engaging and empowering research practices.

### 2.2. Participant Co-Researchers

A total of 8 CYP-parent dyads took part in the study. These comprised 8 CYP with 22q aged between 8 and 30 years old (4 males; 4 females) with current or previous experience of mainstream primary or secondary education, or both, and their parents (all mothers, bar one father). (See Table 1 for CYP demographic data.) Participants were recruited through purposive sampling; calls for expressions of interest were disseminated via relevant third-sector organisations and social media. Recruits were screened by author 3 for their suitability before being invited to join the study, specifically to ensure that inclusion criteria (CYP had a confirmed 22q11.2 deletion diagnosis) and exclusion criteria (CYP and parents with moderate-to-severe learning difficulties and/or with a current psychotic symptom) were met. Pseudonyms were used to maintain participant confidentiality.

### 2.3. Profile of CYP with 22q

CYP participant co-researchers had a primary diagnosis of 22q which affects approximately 1 in every 2000 live births (Blagojevic et al., 2021). Despite being the most common genetic condition after Down’s Syndrome (McDonald-McGinn et al., 1995), it remains relatively unknown outside of specialist services and the individuals and families affected. The condition is highly variable but often associated with distinct facial features and physical health complications from infancy including palatal abnormalities, congenital heart disease and immunodeficiency (McDonald-McGinn et al., 2015, 2022). Individuals with 22q often have developmental delay and mild-to-moderate intellectual disability (Fiksinski et al., 2022) and attend mainstream school or special educational provision, depending on severity. One UK survey (Reilly & Stedman, 2013) found that 76 per cent of 22q children attended mainstream schools. Other (non-UK) evidence suggests this pattern is typical at the primary level and for secondary and further education, special schools are more common (Mosheva et al., 2019). Due to their complex learning profiles, pupils with 22q may struggle in the classroom and are more likely to develop neurodevelopmental conditions such as autism and attention deficit hyperactivity disorder (ADHD) (Cutler-Landsman, 2020). 22q is also highly associated with a range of mental health difficulties including mood disorders, anxieties and phobias (Schneider et al., 2014). Other evidence suggests that some mood and anxiety issues are linked to developmental milestones which require increased independence and social integration (Fabbro et al., 2012; Tang et al., 2015). To the best of the current authors’ knowledge, no prior research has been undertaken with CYP with 22q to explore their experiences of mainstream schooling and wellbeing support.

### 2.4. Procedure

The ethical approval for the study was provided by the University of Roehampton ethics board. In accordance with PAR principles and the affirmative framework, the research involved two interrelated phases. Phase 1 comprised a steering group (SG) meeting (1.5 h duration) with one young person-parent dyad and authors 1 and 3 to establish the best methodological approach for the study. A semi-structured interview technique was agreed, and the SG discussion helped to shape the interview guide, for example, the type of questions and appropriate language, as well as the use of vignettes and visual prompts. The SG meeting took place online and was audio-recorded. Implementing phase 1 ensured that CYP and parents were engaged early in the research process and prompted greater flexibility in phase 2 (knowledge production) through a better understanding of how to accommodate CYP’s different developmental levels and capacities. The current authors were mindful that adult researchers must be prepared to modify their methods and adapt activities and instruments suitably, recognising that the research relationship with CYP is fluid, negotiable and unpredictable (Madge et al., 1997).

Phase 2—the knowledge production phase—was facilitated by authors 1 and 3 and consisted of semi-structured interviews, used to allow the “discovery or elaboration of information that is important to the participant” (Gill et al., 2008, p. 291). Of the 8 CYP-parent dyads, 7 CYP chose to have their parent present during their interviews. Subsequent parent-only interviews were conducted; parent-only data were collected from one dyad (Freya, 8). An interview protocol and question guide (developed from phase 1) were used to navigate the semi-structured interviews which were approximately 45 min in duration; breaks were taken on request. Authors 1 and 3 remained reflexive throughout phase 2 and conscious of disparities in power and status between the adults (researchers and parents) and CYP and employed strategies to address this. For example, regular member checking was performed to prioritise CYP voices throughout. All interviews took place online and were audio recorded. Where possible, parent and CYP interviews took place on the same day to minimise disruption to family routines. Each family received £30 by bank transfer as a token of thanks for their time and contribution to the study. Interview recordings were transcribed by the project’s research assistant (author 2).

### 2.5. Analytical Approach

A thematic analysis (TA)—recognised as theoretically flexible and unconstrained (Braun & Clarke, 2019) was the selected technique. Participatory approaches vary with regard to methodological choices and CYP’s level of involvement. Some authors (Shamrova & Cummings, 2017) have argued that CYP are often disengaged from the analysis stage of research, while A. Xu et al. (2020) have emphasised the active role of the adult researcher in constructing and interpreting realities from meanings through the iterative and reflexive process of TA. In line with a participatory ethos, the current authors avoided prescriptive assumptions in favour of an organic research process that valued and validated the perspectives of CYP and their parents. During the analysis stage, this was facilitated by an inductive/deductive hybrid TA (Fereday & Muir-Cochrane, 2006) which afforded value to the voices of all participants through inductive and “in vivo” coding, while a deductive component, using pre-ordinate categories developed from the literature, enabled greater rigor by virtue of mutual, complementary reinforcement (Proudfoot, 2023). In sum, a predominantly inductive approach and open-coding ensured that respondent meanings were accentuated, while the deductive component meant that the generated themes, well grounded in the data, fully addressed the research aims (Byrne, 2022).

A well-established six-stage process (Braun & Clarke, 2006) guided the analysis. Although the analytical steps are presented in a logical, sequential order, TA is not a linear process but iterative and recursive (Braun & Clarke, 2019). Analysis was conducted manually to sustain sensitivity to the data and allow “a slower and more meaningful interaction with the data” (Maher et al., 2018, p. 11):

- Step 1—familiarisation—began during the data gathering phase (authors 1 and 3) and the transcription process (author 2). Formatted transcripts were shared with all three authors to read/re-read and become highly familiar with the content. Audio recordings were accessible to review for a greater contextual understanding of the data. Initial trends and potentially interesting excerpts were noted. The transcripts were divided equally between the three authors for Step 2.
- Step 2 comprised the generation of initial codes. As Braun and Clarke (2020) have argued, coding (and analysis) rarely delineates neatly as inductive or deductive, but rather emerges as a combination. Each researcher worked systematically through their allocated dataset. Initial codes were collated with accompanying narrative excerpts as illustrative examples and comprised both “in vivo” and descriptive codes. The authors jointly compared and discussed definitions and examples to ensure that each code pertained to more than one data item, thus assuring mutual validity through data triangulation (Proudfoot, 2023).
- Step 3 involved authors searching for several candidate themes from the set of codes. During this stage, codes were actively combined or collapsed to generate meaningful themes or subthemes. In line with Thomas and Harden’s (2008) guidance on thematic synthesis, each author’s independent identification of candidate themes was followed by group discussion of overlaps, divergences and redundancies.
- Step 4 required a recursive review and refinement of the candidate themes by applying the “compare and contrast” method across the whole dataset (W. Xu & Zammit, 2020). This process helped to ensure that any tentative themes were well grounded and reflected participants’ authentic voice. Consequently, some candidate themes were revised or removed to generate the most meaningful and comprehensive interpretation of the entire dataset.
- Step 5 culminated in the final labelling of superordinate themes and subthemes. According to Byrne (2022, p. 1408), labels should be “concise, informative, and memorable” and serve to both capture attention and communicate the essence of the theme, while Braun and Clarke (2020) suggested the authenticity of using short extracts from data items to punctuate theme labels. Both these recommendations were implemented.
- Step 6—writing up the final analysis—was described as the act of telling stories which are the product of the researcher’s prolonged immersion in the data, deep-level thinking and continuous reflection (Braun & Clarke, 2019). Analytic narratives were presented in a logical and meaningful manner to provide a lucid account of the data (Fereday & Muir-Cochrane, 2006) robustly supported by illustrative extracts.

## 3. Results

The current study sought to understand the experiences of CYP with 22q and their parents in mainstream education: to explore gaps in knowledge and support and help inform more inclusive practice. A hybrid inductive/deductive thematic analysis (Fereday & Muir-Cochrane, 2006) generated five superordinate themes: minding the gaps in school support for learning and wellbeing; my mental wellbeing story; power and influence: relationship dynamics—“Some people have been amazing and lots haven’t”; getting it wrong: failing CYP and families; and getting it right: from surviving to thriving—“I’m ambitious”. The themes are summarised in Table 2 with an example extract and organised under subheadings in the supporting analytical narrative that follows.

### 3.1. Theme 1: Minding the Gaps in Support for Learning and Wellbeing

The first superordinate theme encapsulated gaps in support for pupils’ learning and wellbeing. Three subthemes were elicited. The first—all about the learning but what about my learning needs?—encompassed how “inclusive education” was experienced by a subpopulation of CYP with SEND who should benefit from targeted approaches. Academic progress was typically perceived as the school’s overriding priority. Lucy (26) remembered the scrutiny around her school absences:

*They [school] wondered why I kept having days off ill. But that’s because every time I had a PE day, I would be off the next day because of being so tired, but the main thing they were really asking about was why am I off school.*

Kate’s experience suggested that when pupils were on track academically, they were considered to be managing well in school:

*They were very much focused on the academic abilities of children and because she was very quiet, she almost got missed. She was a quiet girl and on track and because she was achieving academically, they didn’t really see what my worries were. So, they would hear me, but they weren’t really listening.*

(Parent of Kate, 24) (primary)

Carl (30) associated getting academic support in secondary school with those pupils who attracted staff attention by being “naughty”, while he felt his own learning needs were easily overlooked in a mainstream setting:

*There was a guy in my year, he had ADHD. So, he was the one that got the support more. It annoyed me a bit sometimes. That’s probably why I struggled with education more than some people. That was my problem. I should have been a bit more naughty. It’s like I could be in a lesson, for instance, Maths—it looks like I’m doing work, but my piece of paper by the end would be blank because I wasn’t really doing anything. So, sometimes I think I should have gone to like a special school for secondary.*

Non-inclusive practices such as failing to adjust the curriculum appropriately were revealed, exposing negative approaches from staff that were highly detrimental to pupils’ learning experience:

*They were doing the practice exams and he was expected to do the Year 4 papers. They told me he got 60 out of 60 for his Maths and I queried it because he’s never got anything like that. So, I asked to see the paper and I did it with him. He couldn’t do any of it and he said, ‘Oh well, the teacher gave me the answers’. [It was] to help him fit in better, but it doesn’t help his future education for understanding his levels or what he can and can’t do.*

(Parent of Callum, 15) (primary)

Several parents attributed their children’s limited academic progress to ill-considered learning targets which set pupils up to fail:

*The targets weren’t achievable. They needed to be a lot smaller than what they’d set out within those plans. And it didn’t really specify how they were going to do it either. So mostly they [targets] just stayed in place [to the next review] because she’d not achieved them.*

(Parent of Kate, 24) (secondary)

Furthermore, common failures to accurately identify and accommodate pupils’ learning needs had repercussions on other aspects of CYP’s school experience including being unfairly disciplined by staff:

*He would get a negative behaviour [mark] because he hadn’t completed or done enough work. Just a vicious circle because he couldn’t do it because he didn’t know what he was doing. He didn’t understand it.*

(Parent of Callum, 15) (secondary)

The second subtheme, awareness and understanding of holistic wellbeing, concerned support for CYP’s physical and socio-emotional needs. Failure to acknowledge and address pupils’ individual needs were highlighted by Freya’s and Callum’s parents, respectively:

*She’s got to walk into the classroom on her own, sit down and put her coat up, on her own. She’s got to deal with that. The TA used to meet us going into school but she doesn’t come anymore because she doesn’t feel [Freya] needs it; but she does.*

(Parent of Freya, 8)

*[Callum] needed to fill his water bottle up... He’s got one great kidney and one not so great, so he is encouraged to always have a bottle of water. So, if he sees it’s empty, it needs to be filled; he hasn’t got the understanding to wait [until after the lesson]. That was an issue [with teachers). Teachers need to understand about his needs, they’re different to the other kids.*

(Parent of Callum, 15)

Parent accounts highlighted how staff were often not attuned to CYP’s socio-emotional needs, nor proactive in supporting them:

*[Freya’s] telling me every day she doesn’t have friends and I said [to the teacher], ‘Can’t you just go over and take her by the hand and just say, ‘Come on, I’ll come with you’ and include her at the table [with other children]. To me, that’s a really common sense, easy thing to do. They totally missed that and said, ‘Well, we can’t make children play with other children’*.

(Parent of Freya, 8)

Conversely, other staff were praised for being highly perceptive and sensitive, knowing subtle signs of distress to look out for in particular pupils:

*At the time, she didn’t feel that she could give any signal to say, ‘I’m not coping.’ Some teachers would recognise the physical change in her which was very subtle. She wasn’t a shouting, screaming, ‘I’m going to take up a lot of space’ kind of child. Her head would go down and she would start picking at her sleeves. So, it was just subtle things that teachers needed to be looking for to really to notice she’s anxious.*

(Parent of Kate, 24) (secondary)

Few CYP had received specific socio-emotional interventions and if a programme had been offered, the potential gains were seen as limited. Only one parent reported that all staff (teaching and non-teaching staff) at her child’s primary school recognised wellbeing as “everyone’s” business, so pupils’ at risk of poor wellbeing were picked up early and action could be taken to support them:

*They [staff] have quite a good awareness, certainly of anxiety. Even the receptionists, they have a chat to her, and they know that she might be feeling anxious. They say, ‘Why don’t we just go and have a little chat’ and then they go and get the [pastoral lead]. They know that she might not actually be physically unwell, it might be something else going on.*

(Parent of Emma, 10)

The final subtheme, invisible 22q?, covered gaps in support uniquely attributed to CYP’s condition. Staff, including SENDCos, had little awareness or understanding of 22q. Pupils without visible manifestations of their condition were less likely to be seen as having additional needs: *“I very much get the impression that this TA [teaching assistant] thinks there’s nothing wrong with her. Because of how she looks physically”* (Parent of Freya, 8). General ignorance of pupils’ condition-related needs was apparent in the lack of sensitivity and inappropriate responses demonstrated by some staff, leaving CYP feeling invalidated and stigmatised:

*There were some not very helpful teachers who didn’t believe I had an issue. They didn’t really believe us in secondary school at all. I had to get actual hand in letters from my doctor to say I have it [22q].*

(Lucy, 26)

For Callum (15): *“[Teachers] were horrible, for example, with toilet passes—they won’t let you go, even though you have one.”* Mis-interpreting condition-related behaviours could result in unwarranted punitive actions: *“When he was walking out of classes because he has to go to the toilet, the teacher saw it as bad behaviour”* (Parent of Callum).

Furthermore, poor awareness and understanding of 22q was linked to negative bias in pupil admissions, as Emma’s parent noted from researching prospective secondary schools:

*With some [SENDCos], I didn’t mention her [22q] diagnosis until a bit later and for others, it was the first thing I said. There was a real difference in the responses. They were a bit, ‘Oh, I wouldn’t want a 22q [child], never heard of that, don’t know what we can do.’ Whereas, as soon as I say, ‘Oh well, you know, she has ASD and a chromosome disorder,’ they will go, ‘Oh, I see, we’ve got X number of children with ASD and these are the interventions that we have. She’s the same child but that opened a box that was not otherwise there.*

(Parent of Emma, 10)

Although families encountered a frustratingly poor understanding of 22q in general, the dearth of “user-friendly” information on such specific conditions and the myriad pressures on school staff were recognised as additional barriers to pupils receiving consistent, well-tailored support in mainstream settings:

[*22q information] doesn’t all make sense to people that don’t have the condition. It’s not very user friendly, but [staff] could have researched the basics, just for a low-level understanding even. But I don’t know, I think when you have that many kids, it’s hard, isn’t it?*

(Parent of Callum, 15) (secondary)

### 3.2. Theme 2: My Mental Wellbeing Story

This superordinate theme comprised CYP’s experiences of mental wellbeing in the school context. The subtheme, triggers and risks: “I felt a bit left out of everything”, identified factors associated with diminished mental wellbeing or inhibitors to healthy wellbeing. For Kate (24), “*When people don’t understand it [22q] it makes me feel different from others.”* Instances of othering and social isolation were widely reported: *“I mainly only had one friend in primary school who also had some sort of disability”* (Lucy, 26). Peer rejection was a common thread in reports of negative social experiences: *“She says they won’t play with her; She said, ‘I say things like what are we going to play now?’, or ‘Do you want to play this or that?’ but they just ignore her.”* (Parent of Freya, 8). Exclusive behaviours extended beyond the school gates and were reinforced, inadvertently or otherwise, by some parents of non-SEND peers:

*[Jack] was not invited to birthday parties. When I look back, it’s quite sad. I think it happened to more [children], as I speak to a lot of people on [22q] groups, and they’ve fed the same things back. They felt their child was excluded, but maybe the parents didn’t know how to deal with a child with disabilities.*

(Parent of Jack, 22) (primary)

Parents reported feeling fearful that their children would be targeted by bullies but had remained ignorant about the level of bullying their children had experienced at the time: “*At secondary school we didn’t realise the extent of the bullying because he held that from us very well. But I was worried”* (Parent of Carl, 30).

Some parents took their own steps to allay their fears and attempted to mitigate the risk of their children being bullied:

*I was terrified of him going to high school because he’s different. I just tried to make him look smart [with designer labels] because I was terrified they [peers] were going to find something extra to bully him for.*

(Parent of Henry, 19) (secondary)

Another important influence on CYP’s wellbeing was related to having a stable school environment. Factors linked to both expected and unpredicted changes in school routines had a negative impact on pupils’ sense of safety, security and comfort:

*I don’t like changes. Like every time you’re moving year. Like say if I was in Year 3 and I was moving to Year 4, I just wanted to stay in Year 3. That’s a change, it won’t be the same.*

(Emma, 10)

*If [Kate] was going to have a substitute teacher, things like that would have really triggered her. So pre-warning that when you go into Maths today, you’ve got a substitute teacher. There was nothing like that, so she would be like a rabbit in the headlights because there was a new strange person in the classroom.*

(Parent of Kate, 24) (secondary)

Major vertical transitions such as the move from primary to secondary school presented elevated risk of anxiety for CYP who were adverse to change and uncertainty: *“She comes to us and she’s already started talking about it [transition to secondary school] and she is very anxious about it. That provoked her most recent visit to the medical room”* (Parent of Emma, 10).

Gaps were identified in the robustness of school policies to fully meet CYP’s needs across the transition process. While some schools had “amazing” plans in place for preparation, they lacked effective follow-up strategies such as supporting CYP’s existing social networks to ensure longer-term transition success: *“They took away a system that was working for her in terms of her anxiety and confidence”* (Parent of Kate, 24). Lack of sensitivity to individual needs, and a “one size fits all” approach was deleterious to more vulnerable pupil’s transition experience and socio-emotional wellbeing:

*I was separated from my primary school friends. They moved on to new friends and I lost friendships. I thought I would be ok, as I’d have people in my class, but I ended up being with no-one [I knew].*

(Kate, 24)

The subtheme, coping mechanisms and support pathways, included different strategies (positive and negative) used to manage school-related stressors and CYP’s experiences of informal and formal support systems. For Emma, school avoidance—creating a pretext to be taken home—was a technique she used to extricate herself from classroom situations she felt difficult to cope with:

*Her way of dealing with it is to decide that she needs to get out of the situation, so she’ll tell someone to tell all the teachers that she’s not feeling well and she needs to go to the medical room which is next to the office. I think her aim from that is that somebody will come and pick her up and take her home.*

(Parent of Emma, 10)

However, more positive ways for Emma to manage stressful events were encouraged at school and at home. For example, she could share her worries by writing them down (and posting them in the class “worry box”). Emma recounted using this strategy to allay her fears about an upcoming trip with the Brownies [youth organisation]:

*I wrote a list about what things I was worrying about [the trip] and we [Emma and mum] took questions to the meeting about the trip. Then [ when I was away] I didn’t even remember my family at home!*

(Emma, 10)

Lucy (26) reflected on how she had evaded lonely break times at secondary school: *“There was an IT lunch group and that helped because otherwise I would just be sitting in the playground on my own.”* For Lucy and other CYP with 22q, having at least one friend created a buffer effect, mitigating the negative impact of peer rejection from wider social groups: *“Abel [my friend] looked after me and helped me”* (Callum, 15) (primary). Nonetheless, much the same as for CYP without SEND, friendships were sometimes fragile: *“[At primary school] they [friends] kind of cared for him, but I do remember times when he would be walking around the edges of the playground just by himself”* (Parent of Henry, 19). The capricious nature of friendship groups can be particularly distressing for CYP who already struggle socially: *“They seem to swap friendship groups a lot and [Freya] struggles if someone’s been a ‘friend’ one day and they’ve had a really nice time, then the next day, they just don’t speak to her”* (Parent of Freya, 8).

### 3.3. Theme 3: Power and Influence: Relationship Dynamics—“Some People Have Been Amazing and Lots Haven’t”

This superordinate theme captured the nature of interpersonal relationships in hierarchical educational settings—specifically those between CYP and staff/professionals and those between parents and staff/professionals, highlighting the powerful influence of these relationships on CYP’s school experiences.

The subtheme, positive relationships: progress and success, encapsulated interpersonal relationships which have shaped pupils’ healthy school experiences, ameliorating both learning and wellbeing outcomes: *“Her German teacher was so good with her and so nurturing, he worked with her one-to-one. They practised the German, and she did her [GCSE] exam just with him”* (Parent of Kate, 24). Having a trusted adult to rely on and go to during the school day was pivotal to CYP’s positive connection to school. As Callum (15) described: *“I can talk to [my teacher] about anything”*, while Lucy (26) reflected on the kindness of individual staff who had helped to make sure her needs were consistently met: *“I did have a lovely Maths teacher who did things for me differently from other kids. He made me my own big book of work that I could do on my own and that was really helpful” (Lucy, 26) (secondary).*

In contrast, the subtheme, negative relationships: barriers and burdens, identified unsupportive or negligent relationships linked to poor outcomes for CYP and their families. For example, the absence of a trusted relationship with one or more member of school staff can inhibit CYP’s sense of belonging to their learning community and hinder their ability to thrive:

*I think he just felt there wasn’t anyone in particular that he could have spoken to at school or that would have even bothered. He said to me, since he never wanted to worry us with all of that, that he just got on with it.*

(Parent of Carl, 30) (secondary)

Although some parents described having positive relationships with at least one member of school staff, these were seen more as the exception, rather than the rule. Several parents reported feeling routinely ignored or not having their views valued and incorporated in decisions related to supporting their children:

[*Primary school staff] would listen, but not a lot. Things would never ever change, and secondary school was worse. Communication was really poor. It never felt like we were working in partnership, that it was coproduction. It always felt like they were the expert. But actually, when it comes to my child, other than her, I am the expert, and I didn’t feel that [understanding] was valued. I just felt like I was an annoying parent and that every time I approached them, it was a bit like, ‘Oh, it’s her again’*.

(Parent of Kate, 24)

### 3.4. Theme 4: Getting It Wrong: Failing CYP and Families

This superordinate theme encapsulated CYP’s and parents’ experiences of being persistently failed. The subtheme, systemic failures, concerned macro-level factors including process issues and the onerous bureaucracy of SEND provision. Despite repeated pledges at a national level for greater collaboration with CYP and parents, families’ experiences were to the contrary:

*I don’t think we were particularly involved enough when they were making the [EHC] plans for her, setting the targets. It was more ‘this is what we’ve come up with and this is what we’re going to do.’ I kick myself because I know so much more now than I did then. And you know, if you get to a review and haven’t achieved the targets and the targets aren’t right, it’s not that the child fails, it’s that the adult has set the wrong targets.*

(Parent of Kate, 24)

An impoverished overall school experience was expressed by Kate: *“I think I mostly just blocked out my time at secondary school from my mind.”*

Parents often felt compelled to be “pushy” to get support in place and expected to encounter adversary at every level of their children’s education:

*We’ve been very pushy about what we want, what she needs put into place [at primary]. I don’t know what it would be like if we weren’t fighting because she needs it all. I think without us pushing and pushing and pushing, we probably wouldn’t have got anything very much. And I think we’ll have the same thing with secondary school”.*

(Parent of Emma, 10)

Some parents felt pressurised into taking the diagnostic route to access additional support: “*I was so relieved to get that [autism diagnosis]…I feel like [staff] do understand it, so they give [a child with 22q] allowances”* (Parent of Freya, 8). Nonetheless, Kate’s parent was sceptical about the positive difference a diagnosis made and had concerns about the potential deleterious effect of labelling on her child:

*We were pushed into it [secondary diagnosis] ___ really did struggle with the diagnosis that she’s got [22q]. She always felt something was wrong with her. And then we said, ‘Well, we’re going to give you another label.’ She was just like, ‘Well, that’s another thing that’s wrong with me.’ But we were told that she couldn’t access the social skills support unless she had a diagnosis. We had to put her through that just to get a six-week course which hasn’t provided any long-term benefits. The diagnosis doesn’t change anything. Parents wait for those two years and think there’s this sudden magic wand to make everything better, but it’s the strategies and support that are important, not whatever label you want to put on it.*

(Parent of Kate, 24)

In addition, the lack of inter-professional consultation and collaboration across the education, health and welfare sectors was a universal concern: *“There’s no joined up [approach] despite the fact that she has her EHCP. There’s no joined up thinking with the speech and language clinic and the same with CAMHS* (Parent of Emma, 10); and *“Very, very rarely [external professional came to review meetings]. It was actually just me a couple of staff and the SENDCo. The medical professionals were invited and didn’t come”* (Parent of Lucy, 26).

Parent frustrations were also keenly felt with respect to the damaging effects of funding deficits on frontline provision which left schools severely under capacity to meet pupils’ unique needs. However, the biggest dilemma facing SEND provision was not generally perceived to be financial. Rather, the lack of the right type of tailored support for CYP during their schooling was considered the biggest problem:

*100% I’d say most of what she needed, wouldn’t have cost them [the government] money. But with the experiences she’s had, from a government perspective, it’s cost them a lot of money because she’s not been able to go through education and get employment as early as she should have. If she’d had the right support, she could have been in employment now.*

(Parent of Kate, 24)

The second subtheme, local culture and climate, centred on how CYP and families had been let down by individual school factors including inflexible practices and policies:

*She’s so polite, a rule follower and hasn’t got a challenging bone in her body. She’s got a lot of sensory difficulties around clothing, so would only wear particular clothing. We had to buy the same shoes every year, same underwear, same socks. She’d been wearing these shoes for a year, but her deputy head saw them and decided they weren’t appropriate and sent her to isolation for the whole day. I tried to explain she’s always worn these shoes and to wear different things causes her a lot of distress, but they didn’t listen.*

(Parent of Kate, 24)

Poor consistency in approach among school staff and lack of sustained support were widely reported: *“When we’re at a meeting and we all seem to be on the same page, that has to continue out of that meeting. But it doesn’t continue and over time it gets complacent and then just disappears”* (Parent of Freya, 8). Ineffective or absent protocols for maintaining parent-school communications and poor internal staff information-sharing were also commonplace:

*Nine times out of 10 when I’ve tried to get a hold of her [the SENDCo], it’s been. ‘She’ll have to ring you back.’ And then reception will e-mail her, so it could take a week. Whatever he’s stressed about needs to be addressed now rather than a week’s time.*

(Parent of Callum, 15) (secondary)

### 3.5. Theme 5: Getting It Right: From Surviving to Thriving—“I’ve Got Ambition”

The final superordinate theme encapsulated good practice and perceptions of what needed to change to improve the school experiences of pupils with 22q and their wider SEND peers. The subtheme, spreading good practice and championing change, highlighted advocacy and action for improving inclusion. Narratives of “getting it right” predominantly related to CYP’s post-school education and early employment experiences. Feeling a sense of belonging to a learning or training community was highly valued by CYP: *“I know you’ll probably laugh at this, but college to me was basically Hogwarts for Harry [Potter]”* (Lucy, 26).

A positive learning culture with ongoing personalised support was linked to successful outcomes: “*[At college] I was able to speak to them [staff] if I was stressed or worried about anything… It was kind of easy to talk to people about my feelings and worries and things”* (Henry, 19). In addition, for Jack (22): *“[At college] I was still doing my Maths and English ‘cos I didn’t pass them in school. I didn’t get it in the first year, but I managed to get it in the second year.”* Furthermore: “*The tutors were brilliant. He went through stages where he wasn’t doing great and didn’t want to carry on. But they talked him through it, and said, ‘it’s OK to struggle; It’s fine.’ And he carried on and passed the course”* (Parent of Jack).

Non-academic classes and tailored support for preparing for young adulthood helped to nurture YP’s burgeoning independence and readiness for future transitions: *There was that sense that they were looking after them [CYP] in _________ [college]. They were so dramatically different to school. The life skills course in college was the best thing for her, definitely. There should be more of it around* (Parent of Lucy, 26).

The subtheme, re-imagining inclusive practice: “I’m just me—that’s the way it is”, embodied strategies which encouraged greater understanding and empower CYP and their families. For Callum (15), schools should *“Help students out when they need it and understand when [I’m] ill and my condition”.* Moreover, a socially affirmative lens around SEND champions a culture that celebrates difference and values listening to the real “experts”. For Lucy (26), prompting this shift tentatively began at secondary school with an idea she came up with:

*I got the courage to talk my science teacher in year nine and said, ‘I have just learnt about my condition, which has to do with chromosomes, can I do presentation about it?’ I ended up doing the presentation and some of the other teachers came in as well to listen to me.*

Nonetheless, beyond raising awareness, securing the shift to an affirmative SEND model and cultural change in mainstream schools (and other settings) can be highly challenging, as Lucy reflected: *“After I had done my first presentation, I think some of the students slightly understood me a bit better. But still, didn’t really want to get to know me”* (Lucy, 26).

Parents were identified as a valuable but underutilised resource in educational settings—capable of sharing knowledge and understanding of specific SEND conditions beyond the confines of specialists and those families immediately affected: *“We did a presentation about 22q to about 12–15 of the tutors, primarily because they didn’t know much about it. So at least they knew more before [Lucy] actually started”* (Parent of Lucy, 26).

In a related domain, forging parent support networks functioned as a catalyst for positive action and change:

*I do parent workshops on all the things I wish that parents knew and wish I’d known. Things around what they should expect from a [EHP] plan and targets and progress. Not going to a meeting to hear that your child is ‘managing well’—they shouldn’t be ‘managing’, they should be making progress. We believe everything professionals tell us and they’re not always right.*

(Parent of Kate, 24)

Belonging to an inclusive and accepting SEND community was important for sharing positive experiences and supporting parents’ own emotional wellbeing:

*When I posted things on the group chats—a milestone like passing his GCSE’s—parents said, ‘I’m so glad you’ve posted that’ because they’re in a place where I was, thinking that their child is not going to get past that stage. These groups are vital, anyone with a child with a disability, whatever it is, should join because they can see from other parents what their kids can do.*

(Parent of Jack, 22)

Conversely, this type of affirmative outlook found on parent forums contrasted sharply with the kind of predictions some parents had received in conversations with professionals:

*[The specialist] surprised us in a lot of things we were told [Callum] wouldn’t be able to do. We were told he couldn’t ride a bike, and you probably won’t be able to have a full conversation with him. And he’s proved everyone wrong.*

(Parent of Callum, 15)

## 4. Discussion

There is general consensus that the SEND system in England is broken (Atkinson et al., 2024; ISOS Partnership, 2024). Findings from the current study do not dispel that proposition. Rather, in line with previous research multiple instances of pupils’ needs being missed or poorly met were identified. Authentic insights from CYP with 22q and their parents highlighted specific gaps and failings in mainstream schools which continue to fall short in their duty to provide inclusive education. Voices included pupils currently in the education system, young people with previous experience and parents. This breadth of contributors enabled unique insights into SEND provision in mainstream schooling in England through the period of reforms introduced a decade ago in the wake of the Children and Families Act 2014 which had envisaged improved “broader outcomes” for CYP (Lindorff, 2020) and more inclusive practice.

### 4.1. Persistent Gaps in Support for Learning and Wellbeing

Atkinson et al. (2024) highlighted a lack of attention to SEND and inclusion in staff training and a curriculum geared to achieving high-stakes qualifications. This predicament renders CYP who do not “fit the mould” of mainstream education and those who require more tailored provision at a structural disadvantage, reflected in the stubborn gap between the learning outcomes of pupils with SEND compared to their non-SEND peers at every level of education (HM Government, 2022). Many pupils in the current study felt unsupported by their teachers and either misunderstood or invisible. Wider research by McPherson et al. (2023) concluded that a high proportion of secondary schools employ teaching methods that many pupils experience as alienating and stressful, particularly those with SEND. This assertion was supported in the current study which revealed poorly set learning targets, which hindered academic progress and set CYP up to fail. Moreover, such a narrow focus on academic attainment can undermine pupils’ perceptions and experiences of inclusion and is detrimental to their mental wellbeing (Boesley & Crane, 2018).

Schools are generally recognised as pivotal settings for promoting and supporting pupil wellbeing (Mansfield et al., 2021) with mandatory responsibilities embedded in the school curriculum (DfE, 2019). A focus on wellbeing in schools fits within the notion of “broader outcomes” for pupils as envisioned in the landmark 2014 reforms (Boesley & Crane, 2018). However, in the current study, despite some evidence of good practice, support for pupils’ holistic wellbeing was patchy, within and between schools and at primary and secondary level. CYP’s wellbeing needs were often overshadowed by the dominant focus on learning outcomes, or otherwise, not identified—as pupils were more likely to internalise their difficulties—and overlooked. Research indicates that a primary SEND diagnosis itself may inadvertently obscure other issues, including socio-emotional difficulties due to diagnostic overshadowing (Children and Young People’s Mental Health Coalition, 2019). In the current study, gaps in support were specifically linked to poor awareness and understanding of CYP’s 22q condition, exacerbated by a perceived reluctance among some staff to learn more about it. McPherson et al. (2023) highlighted the significant pressures teachers face in a predominantly academic-orientated culture and the subsequent detrimental effects this can have on pastoral and additional educational support. Moreover, extra support is required across an increasingly wide spectrum of need, while teachers have reported a lack of clarity in their role and feeling inadequately trained to cope (Education Support, 2023; Hurry et al., 2021).

In line with previous research (GOV.UK, 2021), the current findings showed that pupils’ experiences of SEND support varied widely both within and between schools. This was often attributed to the nature of key relationships between staff and CYP and their parents. When pupils felt that staff understood their needs and were supportive, both learning and school wellbeing improved. Conversely, when relationships were poor or negligible, pupils felt invalidated or neglected and this negatively impacted on their perceptions and experiences of school. These findings add to the evidence depicting a fragmented national landscape of SEND support (Atkinson et al., 2024; ISOS Partnership, 2024; Newlove-Delgado et al., 2021), mirrored in real-world outcomes for CYP.

The SENDCo is usually the main point of contact in schools and leads on securing appropriate support for pupils with additional needs. Again, for CYP and parents in the current study, experiences were mixed, as were perceptions of professional expertise. M. D. Smith and Broomhead (2019) described how families often considered SENDCos as “experts” while, conversely, many staff in the role reported feeling ill equipped to manage their responsibilities. Notably, several parents in the current study agreed with this disappointing assessment due to their personal encounters with SENDCos. They reported having often felt ignored or dismissed, and hierarchical power structures had reinforced feelings of subservience and disempowerment. Dunleavy and Sorte (2022) catalogued parents’ experiences of discrimination in various forms including indifference, gaslighting and social dominance. Chronic lack of capacity for continued professional development (CPD) for education staff has been flagged as a major barrier to ensuring more inclusive practice across settings. Initial teacher training in England offers limited coverage of SEND. To address this gap, Atkinson et al. (2024) insist that CPD on SEND should be mandatory for all educational professionals.

### 4.2. Recognising and Addressing Wellbeing: A Preventative Approach

Research has shown that CYP with SEND are at elevated risk of poor mental wellbeing and were disproportionately affected by the COVID-19 pandemic (Ashworth et al., 2024; Newlove-Delgado et al., 2021; Skrzypiec et al., 2015; Swift et al., 2021). Moreover, Angkustsiri et al. (2014) found that pupils with 22q, as with other SEND groups (Anti-Bullying Alliance, 2023), were more likely to be bullied or excluded by their non-SEND peers. The current findings align with previous research (Goldan et al., 2022; Swift et al., 2021), showing that academic challenges, stigmatisation and peer exclusion place CYP with SEND at higher risk of poor wellbeing. CYP in the current study reported common experiences of peer rejection and social isolation, particularly at secondary school. Condition-related issues such as fatigue and communication difficulties, alongside medical-related absences, added to the challenge of “fitting in” with mainstream peers and developing a sense of school belonging. Conversely, healthy wellbeing is associated with social support and personal factors such as self-esteem and a positive self-image (Gaspar et al., 2016). For CYP in the current study, having at least one friend in school acted as a buffer against the negative impact of wider peer rejection and boosted subjective wellbeing. Friends can offer support through emotional and academic challenges such as adjusting to changes and transitions (Juvonen et al., 2012). Notably, friendship groups typically consisted of other SEND pupils. Clearly, more needs to be done to nurture social and personal skills for pupils with SEND and promote a genuinely inclusive whole school approach to wellbeing. Findings from the current study show multiple instances of missed opportunities, for example, in developing effective and ongoing support for transitions—both more routine changes, such as introducing a new TA, and major upheavals, including, moving from primary to secondary school.

According to the SEND information and advice service SENDIASS (2024), mental wellbeing support for CYP with additional needs is overwhelmingly reactive rather than preventative, and there was limited evidence of proactive approaches in the current study. This may, in part, be linked to wellbeing issues being easily overlooked or mis-diagnosed (Cerebra, 2023). Pertinently, several authors have argued (Kutcher et al., 2015; Miller et al., 2019) that educator mental health literacy is the most important factor for improving pupil outcomes. Other studies have revealed teachers’ high level of concern for pupils’ mental wellbeing, despite lacking confidence in their own ability to provide appropriate support (Maclean & Law, 2022). To improve school-based provision, Norwich et al. (2022) have proposed a dual-factor approach for school settings. This offers a two-dimensional framework representing the distinctive but related aims of school education: wellbeing promotion—fostering subjective wellbeing and enabling pupils to thrive—and mental health services—implementing preventative interventions, supporting those experiencing moderate difficulties and sign-posting those with high needs to professional services. The authors emphasise the vital role schools play in promoting holistic wellbeing through a tiered approach. Specifically, school-wide social emotional learning can be embedded through a whole-curriculum that considers what is to be learned and how it is taught, thus recognising the intrinsic inter-relationship between learning and wellbeing (Lindorff, 2020). In line with this approach, Atkinson et al. (2024) posit that robust pedagogical practice should include making a holistic assessment of CYPs’ needs a standard procedure across education levels, starting with the early years. Although academic abilities are routinely assessed throughout schooling, non-academic abilities are not. Evaluating both academic and non-academic factors would facilitate a better understanding of individual pupils’ holistic needs and generate greater opportunities for intervention to ensure all pupils thrive.

### 4.3. Insights from PAR: Highligting Stubborn Failures and Driving Change

Findings from the current study exposed significant systemic failings with detrimental long-term implications for CYP and their families. In line with the recent SEND review literature (Atkinson et al., 2024; ISOS Partnership, 2024), widespread failure to identify and respond to CYP’s needs in a timely and appropriate way was identified. Delays in obtaining secondary diagnoses and adequate EHCPs left pupils, in some cases for years, without appropriate provision in place. The quality and robustness of plans was often disputed, mirroring other studies which have reported a litany of concerns with EHCPs including vague, generic, and inaccurate content, lack of specific recommendations and unachievable targets (Keville et al., 2024; National Autistic Society, 2021).

A common concern raised by parents related to siloed working practices—both internally in schools and among external professional services. A culture characterised by lack of information-sharing and poor communication between key stakeholders was seen to contribute to delays in diagnoses and support. Despite historic pledges from the government (HCEC, 2019; HM Government, 2023) to make processes more accessible and attuned to pupils’ needs, families’ experiences were often to the contrary. Several parents in the current study used adversarial language such as “constant fighting”, “struggling” and “parent burn out” to express their negative experiences of dealing with services. Recent polls underline escalating levels of dissatisfaction in the national SEND system, for instance, in one 2024 survey involving 52 families, 92 per cent of respondents claimed that engaging with SEND services had been actively detrimental to their mental health (S. Smith, 2024). The legacy of decade old reforms is a SEND system which appears more fraught and adversarial than ever with appeals to the SEND Tribunal rising by 334% between 2014/15 and 2022/23, and tellingly, more than 90 per cent of cases upheld (IPSEA, 2024).

Inevitably, the impact of austerity and funding deficits has been severe for many families. Interestingly, for parents in the current study, financial resources were not considered the primary change needed to improve the school experiences of pupils with SEND. Instead, process issues, such as better information-sharing, collaboration and genuinely listening to families, were seen as paramount for addressing failings, getting the right support in place and managing cost efficiencies in the longer-term. Fundamentally, a paradigm shift towards an affirmative framework for SEND (S. E. Jones, 2024) is urgently needed, whereby CYP’s strengths (as well as needs) are recognised, thus contributing to a more holistic understanding of the support required to improve pupil outcomes and for CYP to thrive. In line with an affirmative framework, positive awareness of SEND contributes to a more inclusive school environment, dispelling stigmatisation and fostering CYP’s sense of belonging rather than marginalisation (Dunleavy & Sorte, 2022).

Previous evidence (Dimitrellou & Male, 2019; GOV.UK, 2021) and current findings support the critical role played by the real “experts”—CYP and parents—in informing meaningful strategies for inclusion, such as creating support systems to nurture friendships, recognising non-academic achievements and raising awareness and understanding of needs and differences. In line with Dimitrellou and Male’s (2019) findings, CYP in the current study broadly agreed with what makes a positive school experience which includes equal attention and support from school staff and positive relationships with peers and adults. While Dimitrellou and Male rightly concluded that listening to pupils with SEND is a powerful tool for informing more inclusive practice, these “desirable” features of schooling are prerequisites to inclusive mainstream education. School assemblies provide a space for pupils to learn about SEND and celebrate differences, while CYP themselves can be active agents in championing this, as seen in the current study. Embedding SEND voice in provision is central to effective and meaningful change through affirmative action in education (S. E. Jones, 2024). Conversely, disregard for pupil voice diminishes CYP’s autonomy and reduces their motivation to learn (Murray & Cousens, 2020), while promoting feelings of frustration and cynicism (M.-A. Jones & Bibb, 2021).

The SEND Code of Practice (DfE & DoH, 2015) clearly states that opportunities for parents to collaborate and contribute towards their children’s provision should be available. However, minimal practical guidance is offered, and there is evidence that parent voice is often marginalised in consultation and decision-making (Ahad et al., 2022). This is supported by the current study, with parents representing an often neglected and under-utilised resource. Quality, readily-accessible information on SEND relating to specific conditions is often lacking in schools—as demonstrated with 22q—and parents can help fill that critical knowledge gap. Experience-informed resources allow professionals and families to share comprehensive information and increase understanding of SEND more widely (Dimitrellou & Male, 2019). Nonetheless, in the current study, CYP and parents received mixed responses from school staff when they offered to play a proactive role in awareness raising and information-sharing. Clearly, there is some way to go in ensuring that genuine collaboration with both parents and CYP is translated from SEND guidance into real-world practice.

### 4.4. Strengths and Limitations of the Study

A key strength of the current study was its commitment to a PAR methodology (Koch & Kralik, 2006) and an affirmative model of SEND (S. E. Jones, 2024; Swain & French, 2000). Adopting a phased approach enabled CYP and their parents to shape the research process to suit their needs rather than be led by the academic researchers. The shift in power dynamics afforded CYP and their parents authentic voice which, for many, had been lacking in their previous encounters with education and other professionals. Nonetheless, it must be acknowledged that simply facilitating voice does not guarantee elicitation. CYP, especially those with SEND, are not accustomed to having their views sought and may be reticent about sharing their opinions and experiences (Jayman & Quickfall, 2024). Furthermore, although steps were taken to mitigate power differentials—such as inviting CYP to help shape the interview schedule—it is unlikely that they were eradicated entirely. For example, social desirability bias (Paulhus, 1991) may have influenced some responses.

A further strength of the study lies in recruiting both current pupils and those with previous experience in mainstream schooling, thus permitting a unique overview of CYP and parent experiences covering a period prior to the Children and Families Act 2014 up to recent SEND reviews (Atkinson et al., 2024; HM Government, 2022; ISOS Partnership, 2024). Conversely, although this approach charts the trajectory of different families’ experiences of mainstream education, exposing persistent gaps and failings, it does not offer exclusive scrutiny of current perspectives which are arguably the most pertinent for informing future strategies. Furthermore, the current study focussed on a subgroup, CYP with 22q, so does not reflect the spectrum of experiences of the wider SEND population in mainstream schools. Although not generalisable, the findings are tentatively transferable.

## 5. Conclusions

The current study focussed on a subpopulation of pupils with 22q and their experiences of mainstream education, revealing significant gaps in provision which resonate with the wider SEND community. The UK education system sets high standards of achievement and behaviour which are routinely measured across national standards throughout a pupil’s schooling. However, the imposition of age-appropriate development and attainment norms conflict with the ideals of inclusive education (Dunleavy & Sorte, 2022). Moreover, normative concepts of achievement and the primacy of non-SEND standards influence the perceptions and experiences of those CYP who do not fit the mainstream mould. The prevailing deficits-based model in schools perpetuates notions of deindividuation, stigmatisation and stereotyping and internalises benign ableism (Dunn, 2019). Unsurprisingly, the competing ideologies of normative success and inclusive practice can engender an incoherent and fractured learning experience which renders CYP with SEND systemically failed. In public discourse, financial constraints are often seen as the root cause of impoverished learning environments; however, the central contribution of human, cultural and ideological factors in catalysing positive change must not be underestimated.

An affirmative perspective places CYP with SEND at the heart of meaningful reform and seeks to understand what inclusion looks like to them—feeling respected, accepted and valued by their teachers and peers, feeling safe and having a sense of belonging to their school community (Goodall, 2018). Re-imagining inclusion in mainstream education requires vision and commitment, while staff training must be prioritised to ensure that the learning and wellbeing needs of all pupils with SEND are fully met. Early intervention and prevention are imperative and require all key stakeholders—educational professionals, local authorities, health and welfare services, researchers and crucially CYP and their families—to work collaboratively to ensure strategies and initiatives are genuinely co-produced, co-owned and robustly evidence-based.

## Figures and Tables

**Table 1:** Demographic data for CYP.

Pseudonym	Age	Gender	Mainstream Experience
Freya	8	Female	Primary
Emma	10	Female	Primary
Callum	15	Male	Primary and secondary
Henry	19	Male	Primary and secondary
Jack	22	Male	Primary and secondary
Kate	24	Female	Primary and secondary
Lucy	26	Female	Primary and secondary
Carl	30	Male	Primary and secondary

**Table 2:** Summary of themes based on the experiences of CYP with 22q and their parents and their perceptions of support in mainstream educational settings.

Superordinate Theme	Subthemes	Illustrative Quotation
Minding the gaps in school support for learning and wellbeing	“All about the learning” but what about my learning needs?	*“They [pupils] were shouting throughout class and I couldn’t handle it because of my hearing issues. I was coming home crying every day because they were shouting, and I was the only one trying to do my work. But you know, we were all getting told off.”* (Lucy, 26) (secondary)
	Awareness and understanding of holistic wellbeing	*“Sometimes I didn’t really get the support I needed. I just had to go into lessons with different people [staff I didn’t know].”* (Henry, 19) (secondary)
	Invisible 22q?	*“There’s no knowledge of 22q within her school. They know what she needs, not because she’s got 22q, because they know her. Nobody in the education system has heard of it. I say, ‘You know it’s the most common chromosome disorder after Down’s?’ -everyone’s heard of Downs They go, ‘No?’ And I say, ‘Yeah, one in every 2,000 people’ and they’re like, ‘Really’?”* (Parent of Emma, 10)
My mental wellbeing story	Triggers and risks: “I felt a bit left out of everything”	*“The main thing the other kids said [at primary school] when I was trying to hang out or play with them was, ‘We don’t want to play with you because you’re different.’ That’s stuck in my head even til now. I tried to make friends [at secondary school], but once again, they didn’t really like me because of how I was.”* (Lucy, 26)
	Coping mechanisms and support pathways	*“I genuinely think that me childminding half of her class got her through primary school because she had those friendship structures outside of school which supported her inside school—all the way through primary.”* (Parent of Kate, 24)
Power and influence: relationship dynamics –“Some people have been amazing and lots haven’t”	Positive relationships: progress and success	*The SENDCo was there for the whole five years [of school] and we built up quite a good rapport. With [Henry] missing so much school and because of his medical needs, we chatted quite a lot. She definitely took the time to learn about him.”* (Parent of Henry, 19) (secondary)
	Negative relationships: barriers and burdens	*“[Teachers] just see everyone the same. It’s like they read from a manual. I got frustrated with it. I’m not an angry person, but when no one’s listening, you’re going to get frustrated and bring anger out. That’s how I felt sometimes. But I hate being angry because that’s not my personality.”* (Carl, 30) (secondary)
Getting it wrong: failing CYP and families	Systemic failures	*“There were no multi agency meetings between school and CAMHS, and there was only one discussion between the autism service and the SENDCo and me.”* (Parent of Kate, 24) (secondary)
	Local culture and climate	*“Sometimes when I went to base (SEND support) it was closed but no-one told me. I would just find out when I needed [to go]. If they had kept it open that would have helped me, and them [teachers] not making me feel bad for needing to go there.”* (Kate, 24) (secondary)
Getting it right: from surviving to thriving	Spreading good practice and championing change	*“In [secondary] school they were able to support me by giving me a coloured timetable with all the different subjects in different colours.”* (Henry, 19)
	Re-imagining inclusive practice: “I’m just me—that’s the way it is”	*“I did a presentation [about 22q] in year nine and another one in year ten for the school. I got what my school calls, the courage medal, for speaking out loud. It’s very brave. That was my first main achievement at that school.*” (Lucy, 26)

## Data Availability

The original contributions presented in this study are included in the article. Further inquiries can be directed to the corresponding author.

## Note

1 An EHCP is a statutory care plan for a young person (aged up to 25), setting out their educational, health and social needs and the additional support to meet those needs. The special educational provision set out in the plan must be provided by a local authority (Livermore, 2023).

## References

[B1-behavsci-15-00022] Ahad A., Thompson A. M., Hall K. E. (2022). Identifying service users’ experience of the education, health and care plan process: A systematic literature review. Review of Education.

[B2-behavsci-15-00022] Ainscow M., César M. (2006). Inclusive education ten years after Salamanca: Setting the agenda. European Journal of Psychology of Education.

[B3-behavsci-15-00022] Angkustsiri K., Goodlin-Jones B., Deprey L., Brahmbhatt K., Harris S., Tony J. (2014). Social impairments in Chromosome 22q11.2 Deletion Syndrome (22q11.2DS): Autism spectrum disorder or a different endophenotype?. Journal of Autism and Developmental Disorders.

[B4-behavsci-15-00022] Anti-Bullying Alliance (2023). Bullying, school experiences and wellbeing: A picture of pupil experience in England in 2023.

[B5-behavsci-15-00022] Ashworth E., Bray L., Alghrani A., Kirkby J. (2024). ‘Trying to stay afloat’: Education professionals’ perspectives on the impact of the COVID-19 pandemic on children with special educational needs and disabilities. Journal of Research in Special Educational Needs.

[B6-behavsci-15-00022] Atkinson A. L., Papen U., Wood M. L. (2024). A country that works for all children and young people: An evidence-based plan for addressing the special educational needs and disabilities (SEND) assessment and support crisis. Child of the North.

[B7-behavsci-15-00022] Barnes M., Harrison E. (2017). The wellbeing of secondary school pupils with special educational needs. Department for education.

[B8-behavsci-15-00022] Blagojevic C., Heung, Theriault M., Tomita-Mitchell A., Chakraborty P., Kernohan K., Bulman D. E., Bassett A. S. (2021). Estimate of the contemporary live-birth prevalence of recurrent 22q11.2 deletions: A cross-sectional analysis from population-based newborn screening. CMAJ Open.

[B9-behavsci-15-00022] Boddison A., Curran H. (2021). The experience of SENCOs in England during the COVID-19 pandemic: The amplification and exposure of pre-existing strengths and challenges and the prioritisation of mental health and wellbeing in schools. Journal of Research in Special Educational Needs.

[B10-behavsci-15-00022] Boesley L., Crane L. (2018). ‘Forget the health and care and just call them education plans’: SENCOs’ perspectives on education, health and care plans. Journal of Research in Special Educational Needs.

[B11-behavsci-15-00022] Bosanquet P., Radford J. (2019). Teaching assistant and pupil interactions: The role of repair and topic management in scaffolding learning. British Journal of Educational Psychology.

[B12-behavsci-15-00022] Bradwell J., Crawford D., Crawford J., Dent L., Finlinson K., Gibson R., Porter E., Kellett M. (2011). How looked after children are involved in their review process. Child Indicators Research.

[B13-behavsci-15-00022] Braun V., Clarke V. (2006). Using thematic analysis in psychology. Qualitative Research in Psychology.

[B14-behavsci-15-00022] Braun V., Clarke V. (2019). Reflecting on reflexive thematic analysis. Qualitative Research in Sport, Exercise and Health.

[B15-behavsci-15-00022] Braun V., Clarke V. (2020). One size fits all? What counts as quality practice in (reflexive) thematic analysis?. Qualitative Research in Psychology.

[B16-behavsci-15-00022] Byrne D. (2022). A worked example of Braun and Clarke’s approach to reflexive thematic analysis. Quality & Quantity.

[B17-behavsci-15-00022] Cerebra (2023). Mental health in children with rare genetic conditions: A guide for parents and carers.

[B18-behavsci-15-00022] Charlton J. I. (1998). Nothing about us without us: Disability oppression and empowerment.

[B19-behavsci-15-00022] Children and Young People’s Mental Health Coalition (2019). Overshadowed: The mental health needs of children and young people with learning disabilities.

[B20-behavsci-15-00022] Curran H. (2019). “The SEND Code of Practice has given me clout”: A phenomenological study illustrating how SENCOs managed the introduction of the SEND reforms. British Journal of Special Education.

[B21-behavsci-15-00022] Cutler-Landsman D. (2020). Educating children with Velo-Cardio-Facial Syndrome, 22q11.2 Deletion Syndrome, and DiGeorge Syndrome.

[B22-behavsci-15-00022] Department for Education [DfE] (2019). Relationships education, relationships and sex education (RSE) and health. Crown.

[B23-behavsci-15-00022] Department for Education [DfE] (2024). Education, health and care plans.

[B24-behavsci-15-00022] Department for Education [DfE], Department of Health [DoH] (2015). Special educational needs and disability code of practice: 0 to 25 years: Statutory guidance for organisations which work with and support children and young people who have special educational needs or disabilities. Crown.

[B25-behavsci-15-00022] Dimitrellou E., Male D. (2019). Understanding what makes a positive school experience for pupils with SEND: Can their voices inform inclusive practice?. Journal of Research in Special Educational Needs.

[B26-behavsci-15-00022] Dockett S., Einarsdottir J., Perry B. (2009). Researching with children: Ethical tensions. Journal of Early Childhood Research.

[B27-behavsci-15-00022] Doucet M., Pratt H., Dzhenganin M., Read J. (2022). Nothing about us without us: Using participatory action research (PAR) and arts-based methods as empowerment and social justice tools in doing research with youth ‘aging out’ of care. Child Abuse & Neglect.

[B28-behavsci-15-00022] Dunleavy A., Sorte R. (2022). A thematic analysis of the family experience of British mainstream school inclusion: Can their voices inform best practice?. Journal of Research in Special Educational Needs.

[B29-behavsci-15-00022] Dunn D. S. (2019). Outsider privileges can lead to insider disadvantages: Some psychosocial aspects of ableism. Journal of Social Issues.

[B30-behavsci-15-00022] Education Support (2023). Teaching: The new reality.

[B31-behavsci-15-00022] Fabbro A., Rizzi E., Schneider M., Debbane M., Eliez S. (2012). Depression and anxiety disorders in children and adolescents with velo-cardio-facial syndrome (VCFS). European Child & Adolescent Psychiatry.

[B32-behavsci-15-00022] Farquharson C., McKendrick A., Ridpath N., Tahir I. (2024). The state of education: What awaits the next government? Institute for Fiscal Studies.

[B33-behavsci-15-00022] Fereday J., Muir-Cochrane E. (2006). Demonstrating rigor using thematic analysis: A hybrid approach of inductive and deductive coding and theme development. International Journal of Qualitative Methods.

[B34-behavsci-15-00022] Fiksinski A. M., Bearden C. E., Bassett A. S., Kahn R. S., Zinkstok J. R., Hooper S. R., Tempelaar W., McDonald-McGinn D., Swillen A., Emanuel B. (2022). A normative chart for cognitive development in a genetically selected population. Neuropsychopharmacology.

[B35-behavsci-15-00022] Gaspar T., Bilimória H., Albergaria F., Gaspar Matos M. (2016). Children with special education needs and subjective well-being: Social and personal influence. International Journal of Disability, Development and Education.

[B36-behavsci-15-00022] Gill P., Stewart K. F., Treasure E. T., Chadwick B. L. (2008). Methods of data collection in qualitative research: Interviews and focus groups. BDJ.

[B37-behavsci-15-00022] Goldan J., Nusser L., Gebel M. (2022). School-related subjective well-being of children with and without special educational needs in inclusive classrooms. Child Indicators Research.

[B38-behavsci-15-00022] Goodall C. (2018). Inclusion is a feeling, not a place: A qualitative study exploring autistic young people’s conceptualisations of inclusion. International Journal of Inclusive Education.

[B39-behavsci-15-00022] GOV.UK (2021). SEND: Old issues, new issues, next steps.

[B40-behavsci-15-00022] GOV.UK (2023). Academic year 2022/23 key stage 2 attainment.

[B41-behavsci-15-00022] GOV.UK (2024). Academic year 2022/23 key stage 4 performance.

[B42-behavsci-15-00022] Halliday A. J., Kern M. L., Garrett D. K., Turnbull D. A. (2018). The student voice in well-being: A case study of participatory action research in positive education. Educational Action Research.

[B43-behavsci-15-00022] HM Government (2022). SEND review: Right support right place right time.

[B44-behavsci-15-00022] HM Government (2023). Special educational needs and disabilities (SEND) and alternative provision (AP) improvement plan. Crown.

[B45-behavsci-15-00022] House of Commons Education Committee (2019). Special educational needs and disabilities: First report of session 2019.

[B46-behavsci-15-00022] Hurry J., Bonell C., Carroll C., Deighton J. (2021). The role of schools in the mental health of children & young people. British Educational Research Association.

[B47-behavsci-15-00022] IPSEA (2024). SEND review is ‘a wolf in sheep’s clothing’—Government proposals will mean complete overhaul of SEND law.

[B48-behavsci-15-00022] ISOS Partnership (2024). Towards an effective and financially sustainable approach to SEND in England.

[B49-behavsci-15-00022] Jayman M., Quickfall A., Jayman M., Glazzard J., Rose A., Quickfall A. (2024). ‘Hear my voice’: Children and young people in schools and research. The BERA Guide to Mental Health and Wellbeing in Schools: Exploring Frontline Support in Educational Research and Practice.

[B50-behavsci-15-00022] Jones M.-A., Bibb S. (2021). Student voice to improve schools: Perspectives from students, teachers and leaders in ‘perfect’ conditions. Improving Schools.

[B51-behavsci-15-00022] Jones S. E. (2024). Raising awareness isn’t enough: The role of the psychology of education in disability-related justice and inclusion in primary classrooms. Psychology of Education Review.

[B52-behavsci-15-00022] Jordan A., Jones E., Horter S., Snape D. (2022). Educational experiences of young people with special educational needs and disabilities in England: February to May 2022. Office for National Statistics.

[B53-behavsci-15-00022] Juvonen J., Espinoza G., Knifsend C. A., Christenson S. L., Reschly A. L., Wylie C. (2012). The role of peer relationships in student academic and extracurricular engagement. Handbook of Research on Student Engagement.

[B54-behavsci-15-00022] Keville S., Mills M., Ludlow A. K. (2024). Exploring mothers’ experiences of accessing an Education Health and Care Plan (EHCP) for an autistic child attending mainstream school in the United Kingdom. International Journal of Developmental Disabilities.

[B55-behavsci-15-00022] Koch T., Kralik D. (2006). Participatory action research in health care.

[B56-behavsci-15-00022] Kuhn L., Norris I., Sawyer G., Schwendel G., Twist L. (2024). Children and young people’s wellbeing and mental health during the COVID-19 pandemic. NFER.

[B57-behavsci-15-00022] Kutcher S., Wei Y., Morgan C. (2015). Successful application of a Canadian mental health curriculum resource by usual classroom teachers in significantly and sustainably improving student mental health literacy. Canadian Journal of Psychiatry.

[B58-behavsci-15-00022] Leatherman J. M., Niemeyer J. A. (2005). Teachers’ attitudes toward inclusion: Factors influencing classroom practice. Journal of Early Childhood Teacher Education.

[B59-behavsci-15-00022] Lindorff A. (2020). The impact of promoting student wellbeing on student academic and non-academic outcomes: An analysis of the evidence.

[B60-behavsci-15-00022] Livermore A. (2023). Our rights, our voices: Young people’s views on fixing the Mental Health Act and inpatient care. Mind.

[B61-behavsci-15-00022] Lloyd-Evans S., Oenga E., MpofuColes A., Gomma T., Karanja E., Duval S., Zischka L., Woronka R., Cleaver M., Tatys K. (2023). Participatory action research: A toolkit. University of Reading.

[B62-behavsci-15-00022] MacDonald C. (2012). Understanding participatory action research: A qualitative research methodology option. CJAR.

[B63-behavsci-15-00022] Maclean L., Law J. M. (2022). Supporting primary school students’ mental health needs: Teachers’ perceptions of roles, barriers, and ability. Psychology in the Schools.

[B64-behavsci-15-00022] Madge C., Raghuram P., Skelton T., Willis K., Williams J., Women and Geography Study Group (1997). Methods and methodologies in feminist geographies: Politics, practice and power. Feminist geographies: Explorations in diversity and difference.

[B65-behavsci-15-00022] Maher C., Hadfield M., Hutchings M., de Eyto A. (2018). Ensuring rigor in qualitative data analysis: A design research approach to coding combining NVivo with traditional material methods. International Journal of Qualitative Methods.

[B66-behavsci-15-00022] Mansfield R., Humphrey N., Patalay P. (2021). Educators’ perceived mental health literacy and capacity to support students’ mental health: Associations with school-level characteristics and provision in England. Health Promotion International.

[B67-behavsci-15-00022] Mason J., Hood S. (2011). Exploring issues of children as actors in social research. Children and Youth Services Review.

[B68-behavsci-15-00022] McCabe E. M., Davis C., Mandy L., Wong C. (2022). The role of school connectedness in supporting the health and well-being of youth: Recommendations for school nurses. NASN School Nurse.

[B69-behavsci-15-00022] McDonald-McGinn D. M., Driscoll D. A., Bason L., Christensen K., Lynch D., Sullivan K., Canning D., Zavod W., Quinn N., Rome J., Paris Y. (1995). Autosomal dominant “Opitz” GBBB syndrome due to a 22q11.2 deletion. American Journal of Medical Genetics.

[B70-behavsci-15-00022] McDonald-McGinn D. M., Hoffman E., Lairson L. A., McGinn D. E., Zackai E. H., McDonald-McGinn D. M. (2022). 22q11.2 deletion syndrome: Setting the stage. The chromosome 22q11.2 deletion syndrome.

[B71-behavsci-15-00022] McDonald-McGinn D. M., Sullivan K. E., Marino B., Philip N., Swillen A., Vorstman J. A. S., Zackai E. H., Emanuel B. S., Vermeesch J. R., Morrow B. E., Scambler P. J., Bassett A. S. (2015). 22q11.2 deletion syndrome. Nature Reviews Disease Primers.

[B72-behavsci-15-00022] McPherson C., Bayrakdar S., Gewirtz S., Laczik A., Maguire M., Newton O., O’Brien S., Weavers A., Winch C., Wolf A. (2023). Schools for all? Young people’s experiences of alienation in the English secondary system. Young Lives, Young Futures.

[B73-behavsci-15-00022] Miller L., Musci R., D’Agati D., Alfes C., Beaudry M. B., Swartz K., Wilcox H. (2019). Teacher mental health literacy is associated with student literacy in the adolescent depression awareness program. School Mental Health.

[B74-behavsci-15-00022] Moore T., McArthur M., Saunders V. (2013). Young people talk about transitioning from youth detention to the community: Making good. Australian Social Work.

[B75-behavsci-15-00022] Mosheva M., Pouillard V., Fishman Y., Dubourg L., Sofrin-Frumer D., Serur Y., Weizman A., Eliez S., Gothelf D., Schneider M. (2019). Education and employment trajectories from childhood to adulthood in individuals with 22q11.2 deletion syndrome. European Child & Adolescent Psychiatry.

[B76-behavsci-15-00022] Murray J., Cousens D. (2020). Primary school children’s beliefs associating extra-curricular provision with non-cognitive skills and academic achievement. Education 3–13.

[B77-behavsci-15-00022] National Audit Office (2019). Support for pupils with special educational needs and disabilities in England.

[B78-behavsci-15-00022] National Autistic Society (2021). School report 2021.

[B79-behavsci-15-00022] Newlove-Delgado T., Robertson W. K., McManus S., Sadler K., Vizard T., Cartwright C., Mathews F., Norman S., Marcheselli F., Ford T. (2021). Mental health of children and young people in England, 2021. NHS Digital.

[B80-behavsci-15-00022] Norden J. (2024). Just 1% of heads say SEND funding meets pupil needs. TES.

[B81-behavsci-15-00022] Norwich B., Moore D., Stentiford L., Hall D. (2022). A critical consideration of ‘mental health and wellbeing’ in education: Thinking about school aims in terms of wellbeing. British Educational Research Journal.

[B82-behavsci-15-00022] Oliver M. (1983). Social work with disabled people.

[B83-behavsci-15-00022] Parentkind (2020). School closures and coronavirus—SEND/SEN survey.

[B84-behavsci-15-00022] Paulhus D. L., Robinson J. P., Shaver P. R., Wrightsman L. S. (1991). Measurement and control of response bias. Measures of Personality and Social Psychological Attitudes.

[B85-behavsci-15-00022] Proudfoot K. (2023). Inductive/deductive hybrid thematic analysis in mixed methods research. Journal of Mixed Methods Research.

[B86-behavsci-15-00022] Punch S. (2002). Research with children: The same or different from research with adults?. Childhood.

[B87-behavsci-15-00022] Reason P., Bradbury H. (2008). Sage handbook of action research: Participative inquiry and practice.

[B88-behavsci-15-00022] Reilly C., Stedman L. (2013). Supporting children with genetic syndromes in the classroom: The example of 22q deletion syndrome. SFL.

[B89-behavsci-15-00022] Sanderson E. (2024). The impact of bullying on pupils with SEND.

[B90-behavsci-15-00022] Schneider M., Debbane M., Bassett A. S., Chow E. W. C., Fung W. L. A., van den Bree M., Owen M., Murphy K. C., Niarchou M., Kates W. R., Antshel K. M., Fremont W., McDonald-McGinn D. M., Gur R. C., Zackai E. H., van Amelsvoot T., Evers L., Boot E., Shashi V., Eliez S. (2014). Psychiatric disorders from childhood to adulthood in 22q11.2 deletion syndrome: Results from the international consortium on brain and behavior in 22q11.2 deletion syndrome. American Journal of Psychiatry.

[B91-behavsci-15-00022] SENDIASS (2024). Mental health and wellbeing.

[B92-behavsci-15-00022] Shamrova D. P., Cummings C. E. (2017). Participatory action research (PAR) with children and youth: An integrative review of methodology and PAR outcomes for participants, organizations, and communities. Child and Youth Services Review.

[B93-behavsci-15-00022] Sharples J., Webster R., Blatchford P. (2018). Making best use of teaching assistants: Guidance report. Education Endowment Foundation.

[B94-behavsci-15-00022] Sibieta L. (2024). School spending in England: A guide to the debate during the 2024 general election. Institute for Fiscal Studies.

[B95-behavsci-15-00022] Skrzypiec G., Askell-Williams H., Slee P., Rudzinski A. (2015). Students with self-identified special educational needs and disabilities (si-SEND): Flourishing or languishing!. International Journal of Disability, Development and Education.

[B96-behavsci-15-00022] Smith M. D., Broomhead K. E. (2019). Time, expertise and status: Barriers faced by mainstream primary school SENCos in the pursuit of providing effective provision for children with SEND. SFL.

[B97-behavsci-15-00022] Smith S. (2024). Parent carers protest as 92.3% say engaging with their local SEN Team was “actively detrimental to their mental health.” Special Needs Jungle.

[B98-behavsci-15-00022] Swain J., French S. (2000). Towards an affirmation of disability. Disability & Society.

[B99-behavsci-15-00022] Swift A., Iriarte E. G., Curry P., McConkey R., Gilligan R., Antunes M. (2021). How disability and other socio-economic factors matter to children’s socio-emotional outcomes. Results from a longitudinal study conducted in Ireland. Child Indicators Research.

[B100-behavsci-15-00022] Tang K. L., Antshel K. M., Fremont W. P., Kates W. R. (2015). Behavioral and psychiatric phenotypes in 22q11.2 deletion syndrome. Journal of Developmental & Behavioral Pediatrics.

[B101-behavsci-15-00022] Thomas J., Harden A. (2008). Methods for the thematic synthesis of qualitative research in systematic reviews. BMC Medical Research Methodology.

[B102-behavsci-15-00022] United Nations General Assembly (1989). Convention on the Rights of the Child.

[B103-behavsci-15-00022] Warnes E., Done E. J., Knowler H. (2021). Mainstream teachers’ concerns about inclusive education for children with special educational needs and disability in England under pre-pandemic conditions. Journal of Research in Special Educational Needs.

[B104-behavsci-15-00022] Woodhead M., Brooker L. (2008). A sense of belonging. Early Childhood Matters.

[B105-behavsci-15-00022] Xu A., Baysari M. T., Stocker S. L., Leow L. J., Day R. O., Carland J. E. (2020). Researchers’ views on, and experiences with, the requirement to obtain informed consent in research involving human participants: A qualitative study. BMC Med Ethics.

[B106-behavsci-15-00022] Xu W., Zammit K. (2020). Applying thematic analysis to education: A hybrid approach to interpreting data in practitioner research. International Journal of Qualitative Methods.

